# Revisiting mechanisms of resistance to immunotherapies in metastatic clear-cell renal-cell carcinoma

**DOI:** 10.20517/cdr.2023.09

**Published:** 2023-05-30

**Authors:** Monica Sheila Chatwal, Jad Chahoud, Philippe E. Spiess

**Affiliations:** Department of Genitourinary Oncology, H. Lee Moffitt Cancer Center and Research Institute, Tampa, FL 33612, USA.

**Keywords:** Metastatic clear-cell renal-cell carcinoma, immune therapy, checkpoint inhibitor resistance, chimeric antigen receptor T-cell therapy

## Abstract

Renal-cell carcinoma (RCC) remains a leading cause of cancer-related mortality worldwide. Though newer therapeutic combinations of immune checkpoint inhibitors and targeted therapies have greatly improved outcomes, resistance to these therapies is becoming a challenge for long-term control. Mechanisms of resistance have been explored in a variety of solid tumors, including RCC. Based upon our review of the current literature on the mechanisms of resistance to immunotherapies for the management of metastatic clear-cell renal cell carcinomas (mccRCC), the ensuing conclusions have been made:

The management of mccRCC has progressed substantially with the advent of checkpoint inhibitors and targeted oral therapies, alone and/or in combination.

Nevertheless, innate or developed resistance to these therapies remains an ongoing challenge, particularly to immune checkpoint inhibitors (ICIs).

Several of the known mechanisms of resistance have been well defined, but recent progression in cellular therapies helps to expand the armamentarium of potential combination options that may overcome these modes of resistance and improve long-term disease control and survival for an otherwise dismal disease.

In the ensuing review and update of the literature on the mechanisms of resistance to immunotherapies in mccRCC, we have revisited the known resistance mechanisms of immunotherapies in metastatic clear-cell RCC and explored ongoing and future strategies to overcome them.

## INTRODUCTION

Renal-cell carcinoma (RCC) remains a common cause of morbidity and mortality worldwide. It is the eighth most common cancer diagnosed in the United States, and in 2022 there were an estimated 79,000 new cases diagnosed, accounting for 4% of new cancer diagnoses in the country^[1]^. Though the relative 5-year survival is nearly 77%, the prognosis of advanced RCC remains dismal with an estimated 5-year survival of 15%^[1]^. Clear-cell renal-cell carcinoma (ccRCC) still accounts for the majority of RCC, with non-clear-cell histologies making up about 25%^[2]^.

Historically, standard chemotherapy and radiation have been ineffective for ccRCC. Clinical studies suggested an immunologic role in the growth and control of RCC, particularly the presence of tumor-infiltrating lymphocytes (TILs) within the tumor and the process of immune evasion^[3-5]^. Immune therapies were then explored as potential therapeutic options, particularly immune cytokines such as interferons and interleukin-2 (IL-2) and later immune checkpoint inhibitors (ICIs). A better understanding of the role of programmed death 1 (PD-1), programmed death ligand 1 (PDL1), and cytotoxic T-lymphocyte–associated protein 4 (CTLA4) led to the eventual use of ICIs in metastatic renal-cell carcinoma (mRCC).

The discovery of a defective von Hippel-Lindau (VHL) gene as a major molecular alteration in the pathogenesis of ccRCC was another leap forward. VHL alterations resulted in upregulation of several growth factors involved in angiogenesis (platelet-derived growth factor receptor-beta, vascular endothelial growth factor [VEGF], and transforming growth factor alpha) using the hypoxia-inducible factor (HIF) pathway. This led to the development of newer therapies specifically targeting these factors^[6]^. VEGF-receptor tyrosine-kinase inhibitors, such as sunitinib, pazopanib, and sorafenib, quickly became the standard of care given their improved response rates, more convenient administration, and manageable toxicity profiles^[7-9]^.

Single-agent use of tyrosine-kinase inhibitors and ICIs was effective but with limited responses and long-term control. Resistance, particularly to ICIs, remains a barrier to achieving and maintaining a durable response to these therapies. It is also important to note that ICIs have several potential adverse effects which must be taken into consideration, especially with long-term use^[10]^. Efforts have been made to combine immunotherapies and anti-angiogenic agents with each other and with other drugs. Other therapies include chemotherapy and radiation for potential synergistic and immunomodulatory effects and to overcome this resistance. Recent reviews summarized potential mechanisms but primarily focused on anti-angiogenic drug resistance^[11]^. In a prior review by Moreira *et al*. published in this journal, resistance mechanisms to immunotherapies in the management of metastatic RCC were explored^[12]^. Here, we revisit these mechanisms and discuss updated ongoing and future strategies for overcoming resistance, particularly adoptive cellular therapies.

### Aim

To review and update the literature on mechanisms of resistance to immunotherapies in mccRCC.

### Methods

Various internet databases were searched, including: PUBMED, Yahoo, Google, and Google Scholar. The search words that were used included: metastatic clear-cell renal-cell carcinoma, immune therapy, immune checkpoint inhibitor, immune checkpoint inhibitor resistance, and chimeric antigen receptor T-cell therapy. One hundred and six (106) references were identified, which were used to write the review and update the literature on the mechanisms of resistance to immunotherapies in mccRCC.

## CURRENT THERAPEUTIC LANDSCAPE

The current treatment landscape for ccRCC in the first and second lines reflects the efficacy seen with immune and anti-angiogenic agents, both alone and in combination, over previously standard cytokine therapies. Choice of treatment is in part led by risk as determined by the International mRCC Database Consortium and Memorial Sloan Kettering Cancer Center/Motzer risk-stratification criteria^[13,14]^. Individual patient factors, including comorbid conditions, concomitant medications, and socioeconomics, also play a large role in the choice of therapy.

Current preferred first-line treatments for favorable risk metastatic ccRCC include: pembrolizumab/axitinib, nivolumab/cabozantinib, and pembrolizumab/lenvatinib, with response rates ranging from 55% to 71%^[15-17]^. Current preferred first-line treatment options for intermediate/poor-risk disease include: pembrolizumab/axitinib, nivolumab/cabozantinib, nivolumab/ipilimumab, pembrolizumab/lenvatinib, and cabozantinib^[18]^.

Second-line treatments vary and include anti-angiogenic and immune-therapy agents that the patient may not have previously received. Inhibitors of the PI3K/AKT/mTOR pathway, such as everolimus and temsirolimus, currently have a role in later lines of therapy^[19,20]^.

## MECHANISMS OF RESISTANCE

ICIs have now become essential in the therapeutic armamentarium for several malignancies, including urothelial carcinoma, melanoma, and non-small-cell lung cancer. In addition to approved combinations for metastatic ccRCC, ICI was recently integrated into the adjuvant treatment of ccRCC with the approval of pembrolizumab post-nephrectomy for high-risk disease, based on results from KEYNOTE-564, noting a disease-free survival benefit of nearly 10% at 24 months, and 30 months disease-free survival HR of 0.63^[21,22]^. Though ICIs have been very effective, we now see evidence of resistance to these therapies, which limits the durability of response.

**Figure 1:**
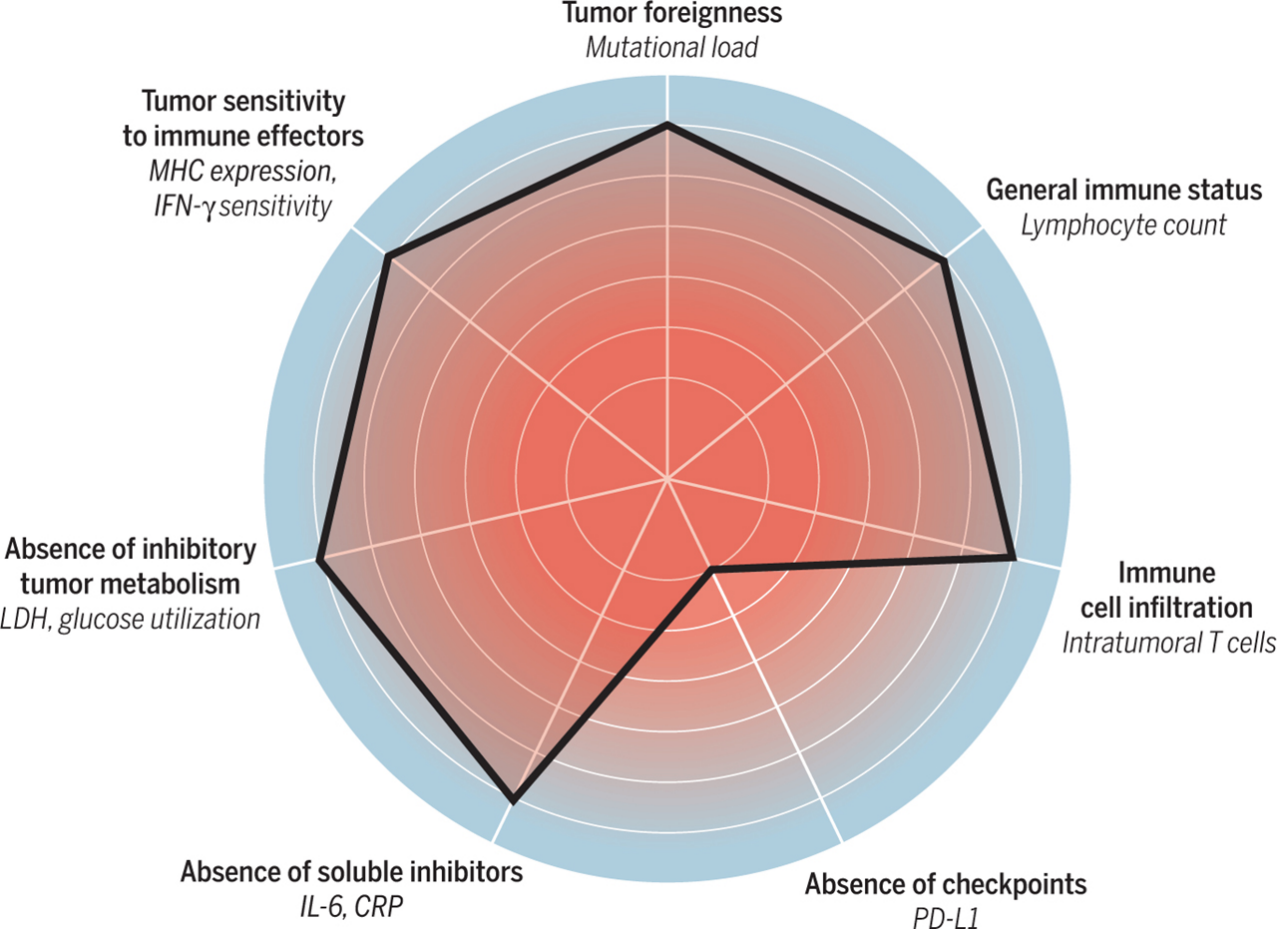
The cancer immunogram - parameters that characterize aspects of cancer-immune interactions where biomarkers have been or may be identified^[23]^. Reprinted with permission from the American Association for the Advancement of Science.

The complex and intricate interaction between the immune system and the cancer cell has been described by the various host- and tumor-specific characteristics that have an impact on this interaction and are visually depicted by Blank *et al.*, where potential and confirmed biomarkers at these levels are also noted [Figure 1]^[23]^. However, this interface is complex, and many parts of this interaction remain undiscovered. In general, immune resistance can be innate, acquired, intrinsic, or extrinsic^[24]^. Innate or primary resistance is an immediate lack of response caused by the presence of resistant clones before starting treatment. Acquired or secondary resistance occurs while on active therapy after an initial response to treatment. Intrinsic resistance occurs when the tumor cells interfere with internal processes, such as cell signaling, gene expression, DNA damage response, and immune recognition, whereas extrinsic resistance occurs through T-cell activation and other processes outside the cell^[24]^. Moreira *et al.* divided potential factors contributing to resistance into three major groups - patient, tumor cell, and tumor microenvironment (TME)^[12]^.

### Patient-associated factors of resistance

Patient-related factors include sex/gender, HLA genotype, sarcopenia, gut microbiome, and antibiotic and corticosteroid use. Recent data have continued to show trends toward sex-related differences in response to ICI, favoring males over females^[25-27]^. Over recent years, there has been tremendous interest in the relationship between the gut microbiome and ICI response. Several studies in melanoma and renal-cell carcinoma have shown that increased diversity in intestinal bacteria is associated with better responses to checkpoint blockade and that certain species may improve or diminish response^[28,29]^. Mouse-model studies exhibited that the microbiome may alter the amount of tumor dendritic cells, antigen-presenting cells, and cytokines^[30]^. Species associated with improved response in mRCC include *Akkermansia* spp. and *Bifidobacterium* spp^[28]^. In a recent phase I study, Dizman *et al*. investigated the use of nivolumab and ipilimumab with or without CBM588, a bifidogenic live bacterial product, in mRCC and found enhanced outcomes among patients receiving combination therapy with the probiotic agent^[31]^. Moreover, antibiotic therapy may alter ICI response because it dysregulates the microbiome. Studies found poor ICI response associated with antibiotic use among patients with mRCC on immunotherapy^[32]^. This was also evaluated in a recent meta-analysis of 10 studies which found decreased progression-free survival (PFS), overall survival (OS), and objective response rate (ORR)^[33]^. In a recent population-based study of patients over 65 years of age, antibiotic use within 1 year of ICI therapy, in particular fluoroquinolones, was associated with worse OS^[34]^. Additionally, steroid use while receiving ICIs has been associated with poor outcomes (PFS and OS). In a recent meta-analysis of 16 studies including non-small cell lung cancer and melanoma, steroid use for supportive care or brain metastases was associated with reduced OS rather than use for adverse effects from ICIs^[35]^. Ongoing studies are evaluating ways of modifying the gut microbiome and factors of antibiotics and steroid use that may be modifiable and affect response to therapy.

### Tumor cell-associated factors of resistance

Tumor cell-related factors for immune evasion and resistance include altered methods of antigen presentation and T-cell exhaustion. By reducing the expression of tumor antigens and downregulating MHC class I, tumor cells may avoid immune surveillance and destruction^[36]^. Alternatively, chronic exposure to an antigen may lead to upregulation of PD-1 or the expression of other inhibitory receptors, such as TIM3, LAG3, BTLA, TIGIT, and VISTA^[24,37,38]^.

This interaction between tumor antigens and immune response led to the theory of combining immunotherapies and stereotactic body radiation therapy (SBRT) as a therapeutic option. Radiation causes tumor necrosis and may release more tumor antigens, which in turn may allow ICIs to work more effectively both locally and potentially beyond what is irradiated. Several studies evaluated the efficacy of this approach, including NIVES, RADVAX RCC, and a high-dose IL2 + SBRT study. NIVES was a phase II study evaluating the role of nivolumab and SBRT in a pretreated patient with mRCC. Sixty-nine patients were enrolled with ORR of 17%, disease control rate of 55%, and OS of 20 months. However, the authors concluded that this combination did not result in improved outcomes among pretreated patients but could be studied further and considered in an oligometastatic population^[39]^. RADVAX RCC evaluated dual-checkpoint blockade with ipilimumab and nivolumab and SBRT. Twenty-five patients were enrolled and initial analysis noted an ORR of 56%^[40]^. Interleukin-2 with SBRT has been studied in metastatic melanoma and mRCC with promising antitumor activity^[41,42]^.

### TME-associated factors of resistance

The TME includes factors extrinsic to the cancer cell and plays a substantial role in regulating T-lymphocytes. The balance between Tregs and Teff cells is an important factor in the response or resistance to ICI, with greater Tregs resulting in a diminished response. Moreover, myeloid-derived suppressor cells are another regulatory mechanism that allows for continued tumor growth through immune regulation^[24]^. Tumor-associated macrophages, TGF-beta, VEGF, and cytokines are all involved in these regulatory processes and may alter the response to ICI therapy.

Chronic antigen exposure can result in upregulation of PD-1 expression and ultimately T-cell exhaustion, a hypofunctional state associated with decreased Teff function^[43,44]^. Exhausted T cells in cancer are similar to those in chronic viral infections, and upregulation of immune checkpoints is a hallmark feature^[45]^. This exhaustion in turn may alter antigen presentation and can be a potential mechanism of resistance to checkpoint blockade in multiple tumor types, including mRCC^[46-48]^.

TILs are strong tumor-defense mechanisms regulating growth and spread. In most malignancies, higher amounts of TILs have been associated with better prognosis and response to ICIs^[49-51]^. However, there are some tumor types in which more TILs are not necessarily better. Though RCC is a heavily T-cell - enriched tumor with high numbers of CD8+ TILs, these are mostly dysfunctional or exhausted^[52-55]^. They also express more inhibitory receptors, such as LAG3 and Tim-3, co-expressed with PD-1, which have been associated with more aggressive phenotypes exhibited by higher TNM staging and a higher Fuhrman grade^[49,56]^.

**Figure 2:**
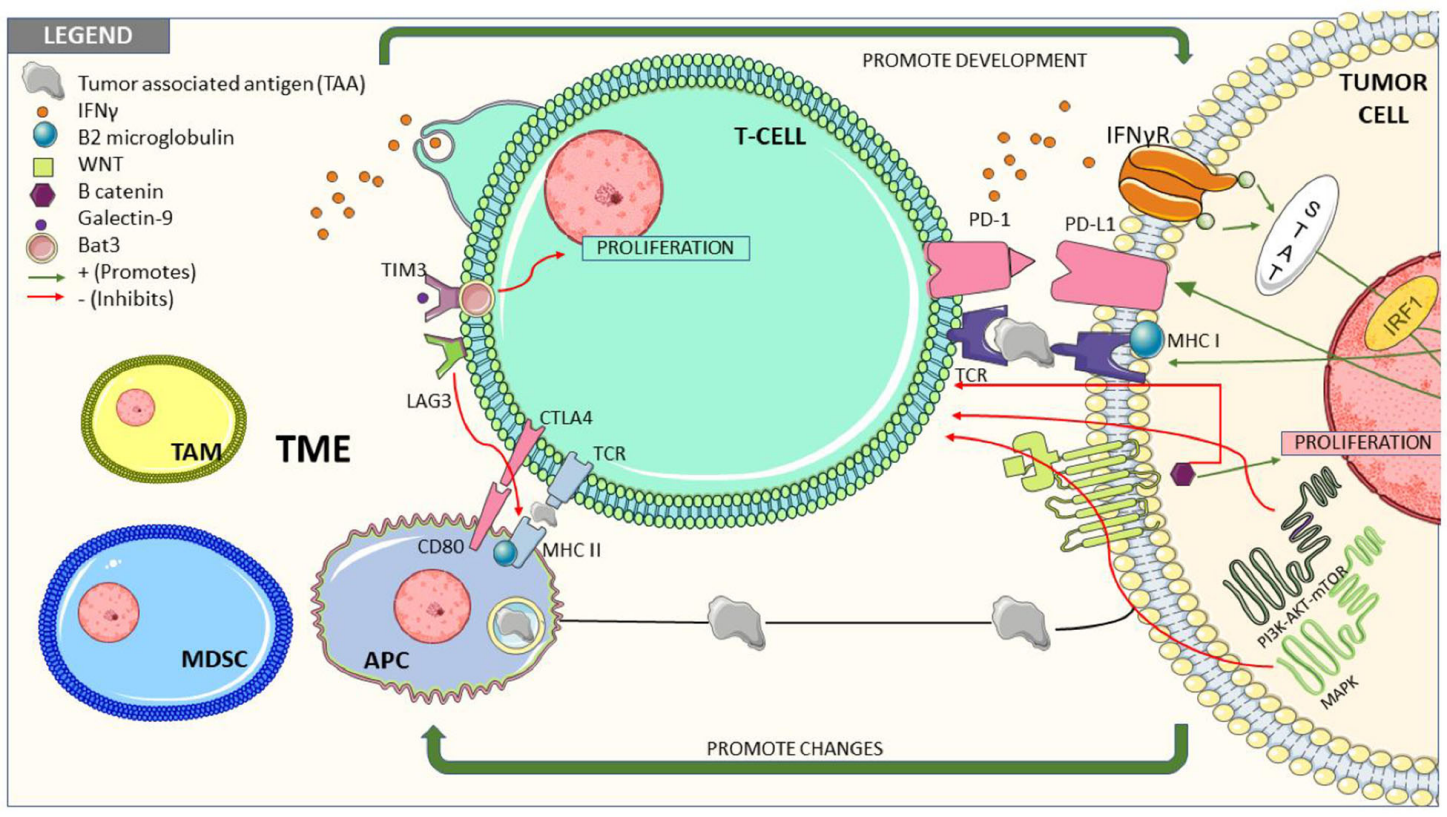
Tumor-intrinsic factors and the TME to describe potential mechanisms of resistance to immune therapies^[61]^. Reprinted with permission from Multidisciplinary Digital Publishing Institute. Abbreviation: TME, tumor microenvironment. Reproduced from^[60]^ under the Creative Commons Agreement License.

Hypoxia is a major feature in the TME of RCC that may contribute to immune dysregulation and tumor progression through several different mechanisms, as was previously described by Moreira *et al*. and others^[12,38,57,58]^. Hypoxia results in the release of hypoxia-inducible factors 1 and 2 (HIF-1a and HIF-2a), which stimulate the expression of inhibitory signals, such as VEGF, CTLA4, and LAG3, and suppress T-cell activation and function^[12]^. Belzutifan, a selective HIF-2a inhibitor, was studied in a phase I trial with promising antitumor activity in heavily pretreated patients with mccRCC^[59]^. It is currently FDA-approved for VHL disease - associated tumors, including RCC, CNS hemangioblastomas, and pancreatic neuroendocrine tumors^[60]^. The use of belzutifan in other settings of RCC and in combination with other therapies, including ICI, is under investigation. Tumor-intrinsic factors and the TME and their interplay are illustrated in Figure 2 as originally developed by Ballesteros *et al*.^[61]^.

## FUTURE DIRECTIONS

Proposed strategies to overcome resistance to ICIs include novel combination approaches, including checkpoint inhibitors with cytokine or chemokine therapy, adoptive cellular therapies, or oncolytic viruses. Biomarkers remain elusive in mRCC as most, including PD-L1, have failed to predict therapeutic response^[62-64]^. Cell-free circulating tumor DNA (ctDNA) may have some utility in mRCC but is limited by lower levels in RCC compared to other solid tumors^[65,66]^. Those with higher tumor volume appear to shed more ctDNA^[67]^. Tumor mutation burden may also be a useful predictor of response to ICIs because a higher tumor mutation burden is associated with a favorable response, though this does not appear to hold for combined checkpoint inhibitors^[68]^. Recently, transcriptomics, metabolomics, and metabolic profiling of RCC cells have allowed for a potential avenue of tumor and resistance detection based on common metabolic features of the cancer cell. Lower OS and greater ICI resistance have been noted in tumor cells with an altered kynurenine/tryptophan ratio and higher hypoxia and Wnt/beta-catenin signaling^[69-72]^.

### Revisiting cytokine therapy

The success of ICIs is limited by resistance reinvigorated interest in cytokine therapy, which had originally been effective in RCC, though without robust responses (around 20%) and their use was limited by challenges in administration and potential toxicities^[73-75]^. In a retrospective analysis, Buchbinder *et al*. evaluated response among patients with metastatic melanoma and mRCC who had received HD IL-2 after prior treatment with PD-1/PD-L1 therapies through the PROCLAIM database. Of the 57 total patients, 17 had mRCC and ORR was 24% with two complete responses^[76]^. In the PIVOT-02 phase I/II study, bempegaldesleukin, a pegylated form of IL-2, was tested with nivolumab as the first line in mccRCC. Initial tumor activity was noted with an ORR of 35% and a complete response of 6%^[77]^. This is being evaluated further in PIVOT-09, the phase III study, though enrollment was recently terminated as of November 2022^[78,79]^. Another recent early-phase trial studied pembrolizumab with HD IL-2 in mRCC and noted an ORR of 70% and no worsening toxicities suggesting further exploration^[80]^.

### Chimeric antigen receptor T-cell (CAR-T) therapy

CAR-T therapy is an adoptive cellular treatment that has revolutionized the management of refractory hematologic malignancies. In essence, a patient’s T cells are removed and modified against a specific cellular target and then infused back into the patient (autologous therapy)^[81]^. This began with the FDA approval of axicabtagene ciloleucel (axi-cel), an autologous anti-CD-19 CAR-T, in October 2017 for relapsed/refractory (R/R) large B-cell lymphoma after two or more prior lines of therapy based on results of the phase I Zuma-1 study^[82,83]^. Since then, axi-cel has been approved for second-line management of large B-cell lymphoma based on results from Zuma-9^[84]^. Other agents and indications have received approval - brexucabtagene autoleucel (brexu-cel) for R/R B-cell precursor acute lymphoblastic leukemia (ZUMA-3); tisagenlecleucel (tisa-cel) for R/R B-ALL (NCT02435849) and R/R large B-cell lymphoma (JULIET); idecabtagene vicleucel (ide-cel) for R/R multiple myeloma (KarMMa); abi-cel for R/R follicular lymphoma (FL) (ZUMA-5); and lisocabtagene maraleucel (liso-cel) for R/R large B-cell lymphomas (TRANSCEND). However, axi-cel’s utility in solid tumors remains under investigation with modest results to date, partly because of tumor-suppressing molecules in the TME and tumor heterogeneity^[85-91]^.

Several preclinical studies assessed potential targets for CAR-T in RCC, with CD70 as a promising site because it appears highly expressed in clear-cell and sarcomatoid tumors^[92-94]^. Early findings from the COBALT-RCC trial (NCT04438083) were recently presented at the 2022 Society for Immunotherapy of Cancer Annual Meeting with safety and clinical activity for CTX130, an allogeneic anti-CD70 CAR-T that has been CRISP/Cas9 gene-edited, in the 13 evaluable patients so far. The ORR was 8% and the disease control rate 77%, with 1 partial response resulting in a CR maintained at 18 months^[95]^. TRAVERSE is an ongoing clinical trial studying the safety and efficacy of ALLO-360, an allogeneic CAR targeting CD70, in mRCC with preliminary data pending (NCT04696731). Another CAR-T product directed against carboxy-anhydrase-IX (CAIX) was promising in preclinical studies, though in an early phase trial, anti-CAR-T antibodies developed with immune responses, and no clinical benefit was noted, and the study was terminated^[96]^. This agent has again been evaluated now in combination with sunitinib, though in mouse models, with some synergistic response seen^[97]^. Ongoing studies are investigating other potential targets and methods of overcoming resistance and suppressive mechanisms of CAR-T by potentially combining it with other agents.

### TIL therapy

TIL therapy has been investigated in mRCC for decades but with limited success, often because of suppressive features of the TME limiting retrieval of large amounts of tumor-reactive TILs^[98-100]^. Figlan *et al*. studied radical nephrectomy with IL-2 and TIL therapy among patients with mRCC with favorable results^[101]^. In a recent commentary, Andersen *et al*. highlighted several prior TIL studies in RCC with variable ORR ranging from 0% to 35%^[99]^. Ongoing studies for TIL therapy are limited though may benefit from combination-based strategies as previously considered, particularly with ICIs to help overcome TME-suppression coinhibitory signals^[102]^. Newer methods of TIL retrieval and expansion are also being explored to help overcome these limiting features^[99,103]^. Table 1 summarizes ongoing cellular therapy trials in mRCC.

**Table 1:** Summary of ongoing cellular therapy clinical trials in metastatic/advanced RCC. Information obtained from clinicaltrials.gov

**Name**	**Identifier**	**Modality**	**Primary Site**	**Status**
Clinical study of CAIX-targeted CAR-T Cells in the Treatment of Advanced Renal Cell Carcinoma	NCT04969354	CAR-T	Affiliated Hospital of Xuzhou Medical University	Recruiting (last updated June 2021)
Clinical study of CD-70 targeted CAR-T therapy in advanced renal cancer	NCT05420519	CAR-T	The Second People’s Hospital of Shandong Province	Recruiting (updated June 2022)
Aldesleukin and pembrolizumab in treating patients with advanced or metastatic renal cell carcinoma	NCT03260504	CAR-T	Fred Hutch/ University of Washington Cancer Consortium	Recruiting (updated October 2022)
TIL Therapy for Metastatic Renal Cell Carcinoma	NCT02926053	TIL	Center for Cancer Immune Therapy, Denmark	Recruiting/ unknown (updated December 2019)
Safety and efficacy of ALLO-316 in subjects with advanced or metastatic clear-cell renal cell carcinoma (TRAVERSE)	NCT04696731	CAR-T	City of Hope, UCLA Medical Center, Moffitt Cancer Center, Memorial Sloan Kettering Cancer Center, Providence Portland Medical Center, MD Anderson Cancer Center	Recruiting (updated March 2022)
P-MUC1C-ALLO1 Allogeneic CAR-T cells in the treatment of subjects with advanced or metastatic solid tumors	NCT05239143	CAR-T	University of California San Francisco, Sarah Cannon Research Institute at HealthOne, University of Kansas Cancer Center, MD Anderson Cancer Center, NEXT Oncology	Recruiting (updated October 2022)
A clinical research about CD70-positive advanced/ metastatic solid tumors treated by CD70-targeted CAR-T	NCT05468190	CAR-T	Henan Cancer Hospital	Recruiting (updated September 2022)
A clinical study of CD70-targeted CAR-T in the treatment of CD70-positive advanced/ metastatic solid tumors	NCT05518253	CAR-T	First Affiliated Hospital, Zhejiang University	Recruiting (updated September 2022)
Safety and Efficacy of CCT301 CAR-T in adult subjects with recurrent or refractory stage IV renal cell carcinoma	NCT03393936	CAR-T	Shanghai Public Health Clinical Center	Active, not recruiting (updated October 2021)
A safety and efficacy study evaluating CTX130 in subjects with relapse or refractory renal cell carcinoma (COBALT-RCC)	NCT04438083	CAR-T	Multiple sites including the United States, Australia, Canada, and the Netherlands	Recruiting (updated May 2022)
HERV-E TCR Transduced Autologous T cells in People with Metastatic Clear-Cell RCC	NCT03354390	TCR	National Institutes of Health Clinical Center	Recruiting (updated December 2022)
Administering Peripheral Blood Lymphocytes Transduced with a CD70-Binding Chimeric Antigen Receptor to People with CD70 Expressing Cancers	NCT02830724	CAR-T	National Institutes of Health Clinical Center	Recruiting (updated January 2023)

### Oncolytic virus-based therapy

Another avenue to overcome resistance is the combination of ICIs with oncolytic viruses, which elicit antitumor immunity^[104]^. Their use seems limited when used alone but may be more beneficial when combined with other agents targeting specific tumor-suppressive signals in the TME. In a phase II open-label study, patients with metastatic melanoma received either talimogene laherparepvec with ipilimumab or ipilimumab alone, and higher antitumor activity with a greater response rate was seen in the combination without increased toxicities (ORR 39%)^[105]^. Recently, a phase I study evaluating NeoVax, a neo-antigen cancer vaccine, with ipilimumab in RCC is actively recruiting (NCT02950766).

### Other targeted approaches

As discussed previously, HIF-2a inhibitors are now being investigated in mRCC, and favorable outcomes led to the approval of belzutifan for VHL-associated tumors, including RCC. The role of HIF-2a inhibitors is being expanded and its use in combination with other agents, including ICIs, is being tested. Preclinical studies for belzutifan used in mouse models without HIF-2a expression found no efficacy for single-agent use, but there was potential synergy with checkpoint inhibitors^[106]^.

Cyclin-dependent kinase 4/6 (CDK4/6) are signaling molecules that promote the progression of the cell cycle by overcoming the tumor suppressor activity of retinoblastoma. Inhibitors of CDK4/6 are currently FDA-approved in the management of breast and ovarian cancers with favorable response rates. Recent preclinical data show promise for CDK4/6 inhibition in RCC to potentiate response with ICIs and chemotherapy^[107]^.

## CONCLUSIONS

The management of mccRCC has progressed substantially with the advent of checkpoint inhibitors and targeted oral therapies, alone and/or in combination. However, innate or developed resistance to these therapies remains an ongoing challenge, particularly to ICIs. Several of the known mechanisms of resistance have been well defined, but recent progression in cellular therapies helps to expand the armamentarium of potential combination options that may overcome these modes of resistance and improve long-term disease control and survival for an otherwise dismal disease.

## DECLARATIONS

### Copyright

**©** The Author(s) 2023.
